# Health-related quality of life in chronic obstructive pulmonary disease patients in Korea

**DOI:** 10.1186/1477-7525-12-57

**Published:** 2014-04-24

**Authors:** Seon-Ha Kim, Yeon Mok Oh, Min-Woo Jo

**Affiliations:** 1 Department of Nursing, Dankook University, 119, Dandae-ro, Dongnam-gu, Cheonan-si, Chungnam 330-714, Korea; 2 Department of Pulmonary and Critical Care Medicine, Asthma Center and Clinical Research Center for Chronic Obstructive Airway Diseases, Asan Medical Center, University of Ulsan College of Medicine, 86, Asanbyeongwon-gil, Songpa-gu, Seoul 138-736, Korea; 3 Department of Preventive Medicine, University of Ulsan College of Medicine, 86, Asanbyeongwon-gil, Songpa-gu, Seoul 138-736, Korea

**Keywords:** Chronic obstructive pulmonary disease, Quality of life, Utility, EQ-5D, CCQ

## Abstract

**Background:**

There are few publications on quality measurement of COPD health state according to the severity level using EQ-5D in Korea. The present study aimed to evaluate the health-related quality of life (HRQoL) in patients with chronic obstructive pulmonary disease (COPD) in terms of disease severity in Korea.

**Methods:**

Totally two hundred patients with COPD were consecutively recruited in one tertiary hospital of Korea. Each respondent was asked to fill out the questionnaire through a face-to-face interview after providing informed consent. The questionnaire included general and clinical characteristics as well as the EQ-5D and Clinical COPD Questionnaire (CCQ). HRQoL was evaluated according to the Global Initiative for Chronic Obstructive Lung Disease (GOLD) criteria and severity of breathlessness.

**Results:**

The adjusted mean EQ-5D index scores were 0.83, 0.88, 0.81 and 0.60 in stage I, II, III and IV, respectively. The EQ-5D index tended to decrease with GOLD criteria. The adjusted mean EQ-Visual Analog Scale (VAS) scores ranged from 65.1 in stage IV to 73.9 in stage I. The CCQ total scores deteriorated with an increasing GOLD stage and severity of breathlessness (all P < 0.001). The correlation between CCQ total score and EQ-5D index was −0.69.

**Conclusions:**

Our study shows that HRQoL in COPD measured by EQ-5D and CCQ worsens with the GOLD stage and severity of breathlessness. EQ-5D and CCQ could be useful instruments for an evaluation of HRQoL in COPD patients in Korea.

## Background

The prevalence and mortality of chronic obstructive pulmonary disease (COPD), already a leading cause of morbidity and mortality worldwide, is increasing [1]. COPD prevalence varies considerably across countries and across different groups within countries, from 3% in India [2] to 19.7% in Uruguay [3]. In Korea, the prevalence of COPD was recently found to be 13.1% in adults over 40 years of age and 31.9% in adults over 65 years of age [4]. The prevalence and mortality of COPD are expected to increase in Korea due to rapid increases in life expectancy. Because COPD is a chronic condition, patient management focuses on improving those symptoms that can adversely affect health status and quality of life [5].

Health-related quality of life (HRQoL) instruments have increasingly been used in clinical trials and health services research, both as primary and secondary endpoints. The EQ-5D questionnaire is a generic, preference-based HRQoL instrument that generates utility scores. These utility scores have been used to compare disease burden across different conditions and to calculate quality-adjusted life years (QALYs) for the economic evaluation of health care interventions [6]. The EQ-5D instrument has been found to be the most frequently used questionnaire in cost-utility studies, including QALY studies [7].

A previous review of the psychometric properties of the EQ-5D supported its construct validity, test-retest reliability, and responsiveness in studying COPD [5]. Another health status measure is the Clinical COPD Questionnaire (CCQ), which is a self-administered instrument that measures clinical control in patients with COPD [8]. The CCQ helps clinicians to identify not only the clinical status of the airways but also activity limitations and emotional dysfunctions in the patients [8]. Also the CCQ is valid, reliable and responsive to changes in patients with COPD [8,9].

The aim of this study was to evaluate the HRQoL of COPD patients in Korea using the EQ-5D and CCQ according to COPD severity. In addition, we explored correlations between the EQ-5D and both disease-specific measures and the forced expiratory volume in one second (FEV_1,_ the volume exhaled during the first second of a forced expiratory maneuver started from the level of total lung capacity).

## Methods

### Study subjects

This study was approved by the Institutional Review Board of Asan Medical Center (approval number: 2011–0119). Two respiratory specialists orally asked their patients with COPD visiting the outpatient clinics of one tertiary hospital to participate in the survey. If the patients wanted to participate in the survey, they were directed to the interview room. All participants provided written informed consent. A total of 202 subjects with COPD were consecutively recruited. One of two interviewers (one is first author, the other is research assistant) performed face-to-face interviews according to a schedule. Two subjects were excluded from analysis because their pulmonary test results were not available. Each respondent was asked to fill out the questionnaire through a face-to-face interview. The survey took approximately 5 minutes to complete. This survey was conducted from 7th May 2012 to 18th July 2012. There was no withdrawal during the survey.

### Information

The questionnaire included general and clinical characteristics, the EQ-5D, and the CCQ. Demographic factors included sex, age, level of education and current smoking status. Clinical characteristic consisted of current symptoms (cough, sputum, chest pain, dyspnea, weight loss, fever, and fatigue), the number of admissions during the previous year, and the severity of breathlessness. Patients were asked to describe their severity of breathlessness as one of three categories that reflected their current state: short of breath during strenuous activities, stopping to catch their breath after a few minutes walking or breathless when dressing or washing.

The EQ-5D is a generic preference-based measure where health status is described in terms of five dimensions: mobility, self-care, usual activities, pain/discomfort, and anxiety/depression. Each dimension has three levels, indicating no problems, some or moderate problems, and extreme problems [6]. The EQ-5D provides a single index value for health status by applying a formula that essentially attaches values to each of the levels in each dimension [10]. The EQ-5D index of health state in this study was calculated using the valuation set of the Korean population [11]. Therefore, the possible range of EQ-5D scores was from −0.171 to 1.0, with 1.0 denoting full health (11111 state) and 0.0 denoting death. The EQ-Visual Analog Scale (VAS) records the respondent’s self-rated health on a vertical, VAS where the endpoints are labeled ‘Best imaginable health state’ (100) and ‘Worst imaginable health state’ (0) [10].

The CCQ consists of ten items divided into three domains: symptoms, functional states, and mental states. Subjects are asked to answer based on their experiences during the previous seven days. Subjects responded to each question using a 7-point scale where 0 = asymptomatic or no limitation and 6 = extremely symptomatic or totally limited [9]. Individual items in the CCQ are equally weighted; thus, the total score and three domain scores are calculated by averaging the related items (ranging from 0 to 6) [9]. A higher score represents a poorer health status.

The severity of COPD was categorized by two different approaches. The first classification of severity was based on the FEV_1_ as a percentage of predicted normal values (FEV_1_% predicted) from the Global Initiative for Chronic Obstructive Lung Disease (GOLD) guidelines: stage I = ≥80%; stage II = 50–79%; stage III = 30–49%; and stage IV = <30% [12]. FEV_1_ values were retrospectively obtained from patients’ medical records. FEV_1_ values measured at the nearest day of survey date including the visit day were used. The second criterion was based on the severity of breathlessness in our questionnaire. It was modified by authors referring to another publication [13].

### Analysis

According to these severity criteria, we calculated both crude means and adjusted least square means of the EQ-5D index, EQ-VAS and CCQ total scores. Analysis of covariance (ANCOVA) was performed to calculate the adjusted mean of each HRQoL score. The criteria of COPD severity were used as the factors and age group, gender, level of education, and smoking status were used as the covariates. The sensitivity of both the EQ-5D index and the CCQ scores according to the GOLD stage were explored using the effect size. The effect size was calculated as the ratio between the mean score differences between two consecutive GOLD stages and the pooled standard deviation (SD, average variation of two groups) [14]. The effect size was interpreted using Cohen’s criteria [15]. While 0.2 was interpreted as a small magnitude of effect, 0.5 indicated a medium effect, and 0.8 was interpreted as a large effect.

To explore the convergent validity of EQ-5D in COPD patients, the correlations between EQ-5D index and EQ-VAS, CCQ, the disease-specific and clinical measures and FEV_1_ were examined using the Pearson correlation coefficient. We expected that there would be a weak correlation between FEV_1_ and a patient’s health related quality of life score, while the correlation between EQ-5D index and CCQ score was moderate to high based on previous publications [8,9,12]. The internal consistency of the CCQ was evaluated with Cronbach’s alpha.

Statistical analyses were conducted with the SAS software package (version 9.1; SAS, Cary, NC, USA). P < 0.05 was considered to be statistically significant.

## Results

A total of 200 out of 202 completed questionnaires were used to analyze the HRQoL. The characteristics of the subjects are shown in Table 1. The mean age of the subjects was 68.5 (SD = 9.1) years and 91.5% were male. Of these, 18 were current smokers and 156 were ex-smokers. Sputum was the most common symptom for subjects followed by fatigue and dyspnea. Fifty percent of subjects reported mobility problems in the EQ-5D. The mean EQ-5D index was 0.84 (SD = 0.16) and the mean EQ-VAS score was 69. The mean values of the lung function was 50.7, 56.3, 78.8 in FEV1/FVC, FEV1 Predicted (%), and FVC Predicted (%), respectively.

**Table 1:** Demographic and clinical characteristics of study subjects

**Variables**	**n**	**(%)**
Age, years		
Less than 60	25	(12.5)
60–69	74	(37.0)
70–79	85	(42.5)
80 and more	16	(8.0)
Gender,		
Male	183	(91.5)
Level of education, years		
6 or below	48	(24.0)
7–9	45	(22.5)
10–12	62	(31.0)
13 and more	45	(22.5)
Smoking status		
Non-smoker	26	(13.0)
Ex-smoker	156	(78.0)
Current smoker	18	(9.0)
Current symptoms		
Sputum	146	(73.0)
Fatigue	117	(58.5)
Dyspnea	115	(57.5)
Cough	99	(49.5)
Chest pain	47	(23.5)
Weight loss	40	(20.0)
Fever	14	(7.0)
Reporting of problems in the EQ-5D dimensions		
Mobility	99	(49.5)
Self-care	43	(21.5)
Usual activities	88	(44.0)
Pain/discomfort	69	(34.5)
Anxiety/depression	66	(33.0)

The EQ-5D index and EQ-VAS scores by COPD severity according to GOLD stage are illustrated in Table 2. The adjusted mean differences in the EQ-5D index and EQ-VAS scores between the four different groups were statistically different (P < 0.001, P = 0.033, respectively). Following post hoc pairwise comparison, the adjusted mean EQ-5D indices in the stage I, II and III groups were between 0.81 and 0.88, which were significantly higher than the 0.60 of the stage IV group (all P values <0.001). The adjusted mean EQ-VAS scores were 73.9 in stage I and 65.1 in stage IV. The effect sizes for the EQ-5D index between stages II and III and stages III and IV were 0.47 and 1.18, respectively, while the corresponding values for the EQ-VAS were 0.33 and 0.19, respectively.

**Table 2:** EQ-5D index and EQ-VAS scores by GOLD criteria

	**Crude mean**	**(SD)**	P value^*^	**Adjusted mean**^**a**^	**(SE)**	**P value**^**†**^	**Grouping**^**b**^
EQ-5D index							
Stage I, n = 13	0.83	0.17	<0.001	0.83	0.04	<0.001	A
Stage II, n = 114	0.88	0.12		0.88	0.02		A
Stage III, n = 59	0.82	0.16		0.81	0.03		A
Stage IV, n = 13	0.61	0.26		0.60	0.04		B
EQ-VAS							
Stage I, n = 13	71.9	18.9	0.042	73.9	5.4	0.033	A
Stage II, n = 114	71.9	17.8		75.1	2.9		A
Stage III, n = 60	65.0	20.6		68.9	3.3		A
Stage IV, n = 13	60.9	13.9		65.1	5.6		A

SE, standard error of the mean.

^*^Analysis of variance (ANOVA) tests of whether the four groups are equal.

^†^Analysis of covariance (ANCOVA) tests of whether the four groups are equal.

^a^Means were adjusted by age group, level of education, gender, and smoking status.

^b^Different alphabet (A, B) means a statistically significant difference group after Bonferroni correction. The same grouping was shown in both ANOVA and ANCOVA.

The CCQ scores by COPD severity according to GOLD stage are shown in Table 3. All scores for the CCQ significantly increased as the COPD stage worsened. In post hoc analysis, the functional state scores for the CCQ in stage II were significantly different from those of stages III and IV (P = 0.003 and P < 0.001, respectively) while the scores between stages I and II were not significantly different (P = 0.680). The effect sizes for the CCQ total score between stages II and III and between stages III and IV were 0.37 and 0.65, respectively, while that for the CCQ total score was 0.20 between stage I and stage II.

**Table 3:** CCQ scores according to GOLD criteria

	**N**	**CCQ total score**		**CCQ domain score**					
				**Symptom**		**Functional state**		**Mental state**	
		**Mean**	**(SE)**	**Mean**	**(SE)**	**Mean**	**(SE)**	**Mean**	**(SE)**
Stage I	13	0.8	(0.3)	1.1	(0.3)	0.6	(0.3)	0.4	(0.4)
Stage II	114	0.9	(0.3)	1.3	(0.2)	0.7	(0.2)	0.8	(0.2)
Stage III	60	1.4	(0.2)	1.6	(0.2)	1.3	(0.2)	1.1	(0.3)
Stage IV	13	2.0	(0.3)	1.7	(0.3)	2.3	(0.3)	2.2	(0.4)
P trend^*^		<0.001		0.026		<0.001		<0.001	

All CCQ scores [adjusted mean values (SE)] were adjusted by age group, level of education, gender, and smoking status. SE, standard error of the mean.

^*^The P–trend value tests the null hypothesis that there is no linear trend between the CCQ means and GOLD stage order.

HRQoL scores according to breathlessness are shown in Table 4. In all HRQoL scores, quality of life significantly worsened as severity of breathlessness increased. The mean EQ-5D index was 0.87 for subjects who felt short of breath during strenuous activity, 0.74 for subjects who had to stop to catch their breath after a few minutes walking, and 0.54 for subjects who were breathless when dressing/washing. In pairwise comparison, all HRQoL score differences by the level of breathlessness were significantly different except for that of the EQ-VAS between levels 2 and 3 (P = 0.061). The effect sizes for the EQ-5D index in breathlessness between the level 1 and level 2 groups and between the level 2 and level 3 groups were 0.55 and 1.14, respectively, while those for the CCQ total score were 0.60 and 1.45, respectively.

**Table 4:** HRQoL scores according to the severity of breathlessness

	**Level 1, n = 146**		**Level 2, n = 45**		**Level 3, n = 7**		P trend^*^
EQ-5D index	0.87	(0.02)	0.74	(0.03)	0.54	(0.06)	<0.0001
EQ-VAS	75.07	(2.63)	65.64	(3.46)	52.57	(7.11)	<0.0001
CCQ total score	0.82	(0.12)	1.89	(0.16)	3.36	(0.16)	<0.0001
CCQ domain score							
Symptoms	1.14	(0.13)	1.97	(0.17)	3.56	(0.36)	<0.0001
Function	0.58	(0.14)	1.96	(0.19)	3.08	(0.39)	<0.0001
Mental	0.65	(0.19)	1.59	(0.25)	3.53	(0.52)	<0.0001

Level 1, short of breath during strenuous activities; level 2, stopping to catch breath after a few minutes walking; level 3, breathless when dressing or washing.

All scores [adjusted mean values (SE)] were adjusted by age group, level of education, gender, and smoking status. SE, standard error of the mean.

^*^The P –trend value tests the null hypothesis that there is no linear trend between the HRQoL scores and severity of breathlessness order.

Cronbach’s alpha was 0.88 for the CCQ total items. Internal consistencies of symptom, functional state and mental state were 0.69, 0.85 and 0.63, respectively. Table 5 shows the Pearson correlation coefficients between the EQ-5D, CCQ and FEV_1_% predicted. Correlation coefficients between EQ-5D index and CCQ instrument ranged from −0.55 on the CCQ symptom score to −0.69 on the CCQ total scores. The correlation between either the EQ-5D index or CCQ total scores and the FEV_1_% predicted was 0.30 and −0.35, respectively.

**Table 5:** **Pearson’s correlation between CCQ, EQ-5D, and FEV**_**1**_**% predicted**

	**EQ-5D index**	**EQ-VAS**	**CCQ symptom**	**CCQ functional state**	**CCQ mental state**	**CCQ total**	**FEV**_**1**_**% predicted**
EQ-5D index	1.00	--	--	--	--	--	--
EQ-VAS	0.41	1.00	--	--	--	--	--
CCQ symptom	−0.55	−0.43	1.00	--	--	--	--
CCQ functional state	−0.64	−0.50	0.64	1.00	--	--	--
CCQ mental state	−0.59	−0.42	0.62	0.64	1.00	--	--
CCQ total	−0.69	−0.53	0.87	0.90	0.82	1.00	--
FEV_1_% predicted	0.30	0.18	−0.22	−0.36	−0.31	−0.35	1.00

All correlations are statistically significant.

## Discussion

The present study shows that the mean EQ-5D index decreased as the GOLD criteria stages II–IV (based on FEV1% predicted) and extent of breathlessness increased. Lower EQ-5D utility scores were associated with very severe COPD. The difference between moderate and severe COPD based on the FEV1% predicted value was one-third of the difference between the very severe and severe states. However, the utility scores of different COPD severities were not significantly different except for very severe COPD. In contrast, based on the severity of dyspnea, the EQ-5D utility score showed significant differences in all group comparisons and the corresponding effect size of patients with different levels of dyspnea ranged from 0.47 to 1.18, which indicated around a medium-to-large effect based on Cohen’s criteria.

Also all CCQ scores significantly increased according to both the GOLD stage and the severity of breathlessness. Our analysis showed that the differences in the CCQ total scores among GOLD stages were more than 0.4 except between stages I and II. Kocks et al. [16] reported 0.4 as the minimal clinically important difference of the CCQ total score. The effect sizes for the differences in the CCQ total scores between GOLD stages II and III and between GOLD stages III and IV demonstrated the discriminative capacity of the CCQ measure. The CCQ items were internally consistent (Cronbach’s alpha = 0.88) and all CCQ scores were also strongly correlated with the EQ-5D index. Thus, our results suggest that both the EQ-5D and CCQ instrument can be valid in measuring HRQoL in patients with COPD in Korea and that the severity of breathlessness is a more distinguishable criterion of both HRQoL instruments than the GOLD criteria.

We compared the present findings with studies from other countries that used the EQ-5D with the UK-based algorithm to measure the utility scores of patients with COPD according to GOLD stage. Rutten-van Molken et al. [17] and Stahl et al. [18] reported mean utility scores of 0.787 and 0.73 for moderate, 0.750 and 0.74 for severe and 0.64 and 0.52 for very severe COPD, respectively. A systematic review [5] reported the following pooled mean utility scores (SD) according to GOLD stage: 0.74 (0.66–0.83) in moderate, 0.69 (0.60–0.78) in severe and 0.61 (0.44–0.77) in very severe COPD. When our data were applied to UK-based algorithm to calculate the utility scores of patients with COPD, mean utility score plummeted to 0.69, 0.58 and 0.24 for moderate, severe, and very severe COPD, respectively.

Differences in utility scores by each COPD state between countries could be due to differently calculated algorithms or subject heterogeneity, in particular in terms of current symptoms and breathlessness severity that is regardless of airflow limitation severity. Substantial differences in the UK and Korean value sets exist and the UK algorithm tends to give lower EQ-5D scores than the Korean algorithm [19]. When we reanalyzed our data using the UK algorithm, the utility weights of all COPD health states were lower than those of previous studies. The CCQ total scores for mild, moderate, severe, and very severe COPD in the Netherlands were 1.0, 1.3, 1.9 and 2.5, respectively [8], and those for mild to moderate, severe, and very severe COPD in Italy were 0.9, 1.4, and 2.6, respectively [9]. As our subjects showed a higher HRQoL in very severe COPD than previous findings, it could mean more well-controlled patients were recruited.

Several studies showed a generally weak association between changes in FEV_1_ and the generic HRQoL [20,21]. Our results also showed a low correlation between the EQ-5D index and the FEV_1_, whereas the results of Stahl et al. [18] showed a strong relationship between HRQoL and an impaired lung function. Even with disease-specific instruments designed to measure the HRQoL in patients with obstructive airways disease, such as the St. George’s Respiratory Questionnaire, the association between the HRQoL and FEV_1_ was weak [21,22].

To the author’s best knowledge, no study has been published yet regarding HRQoL of according to severity in Korean COPD patients. Our study measured HRQoL using EQ-5D and CCQ according to COPD severity and these instruments could be utilized as an outcome measure in an economic evaluation of COPD intervention programs or clinical studies in Korea.

Our study has several limitations. First, subjects were recruited during outpatient visits, reducing the number of patients with very severe COPD patients. As a result, the mean utility score of the very severe COPD state was less precise than that of milder states. Small sample sizes for the GOLD stage 1 may not represent feature in these group as well. However, a review study reported zero difference of pooled EQ-5D utility index between mild to moderate stages [5]. Second, COPD is characterized by fluctuations in symptoms and the prevention of exacerbation is important in COPD management. However, our subjects mainly consisted of stable patients without exacerbation, which means that the results of this study are less useful for patients with exacerbation and that further research is required regarding the evaluation of HRQoL in COPD patients with exacerbation. Third, there was a disproportionate distribution of gender in our study. Among Korean adults over the age of 40 years, 13.4% (men, 19.4%; women, 7.9%) had airflow obstruction [23]. Our data could under-represent female compared to the COPD prevalence by gender. Fourth, only a third of participants measured FEV1 on the day of the interview and others performed pulmonary function test during a previous visit. FEV1 on the interview date could reflect the better quality of life of the day, but changes of FEV1 from a previous visit were not large due to a chronic feature of COPD. As a matter of fact, the findings of the subgroup analysis of the FEV1 that were measured on the day of interview were similar to the original one. Lastly, the severity of breathless in our study could be regarded as being somewhat arbitrary. To evaluate the impact of various symptoms on HRQoL in COPD patients, more standardized criteria (e.g., the modified Medical Research Council questionnaire, the COPD assessment test) could be used.

## Conclusions

In conclusion, our results suggest that both the EQ-5D and CCQ instrument can be valid in measuring HRQoL in patients with COPD in Korea and that the severity of breathlessness is a more distinguishable criterion than the GOLD criteria if both HRQoL instruments are compared. Further research is recommended for estimating utility in exacerbation.

## Abbreviations

ANCOVA: Analysis of covariance; CCQ: Clinical COPD questionnaire; COPD: Chronic obstructive pulmonary disease; FEV1: Forced expiratory volume in one second; GOLD: Global initiative for chronic obstructive lung disease; HRQoL: Health-related quality of life; QALYs: Quality-adjusted life years; VAS: Visual analog scale.

## Competing interest

The authors declare that we have no competing interest.

## Authors’ contributions

All authors contributed to the conception and design of the study, the acquisition of data, and the interpretation of the results. SHK analyzed the data and was involved in drafting the manuscript; MWJ and YMO were involved in revising the manuscript to ensure its critically important content. All authors have read and approved the final.
